# Addressing the important error of missing surgical items in an operated patient

**DOI:** 10.1186/s13584-022-00530-z

**Published:** 2022-04-05

**Authors:** Sergio Susmallian, Royi Barnea, Bella Azaria, Martine Szyper-Kravitz

**Affiliations:** 1 Department of Surgery, Assuta Medical Center, 20 Habarzel Street, 69710 Tel Aviv, Israel; 2 Faculty of Medicine, Ben Gurion University of the Negev, Beer Sheva, Israel; 3 Assuta Health Services Research Institute, Assuta Medical Center, Tel-Aviv, Israel; 4 School of Health Systems Management at Netanya Academic College, Netanya, Israel; 5 Medicine Division, Assuta Medical Center, Tel Aviv, Israel; 6 Patient Safety and Risk Management Unit, Assuta Medical Center, Tel-Aviv, Israel

**Keywords:** Retained surgical items, Never events, Complications

## Abstract

**Background:**

We aim to analyze the characteristics of incidences of missing surgical items (MSIs) and to examine the changes in MSI events following the implementation of an MSI prevention program.

**Methods:**

All surgical cases registered in our medical center from January 2014 to December 2019 were retrospectively analyzed.

**Results:**

Among 559,910 operations, 154 MSI cases were reported. Mean patient age was 48.67 years (standard deviation, 20.88), and 56.6% were female. The rate of MSIs was 0.259/1000 cases. Seventy-seven MSI cases (53.10%) had no consequences, 47 (32.41%) had mild consequences, and 21 (14.48%) had severe consequences. These last 21 cases represented a rate of 0.037/1000 cases. MSI events were more frequent in cardiac surgery (1.82/1000 operations). Textile elements were the most commonly retained materials (28.97% of cases). In total, 15.86% of the cases were not properly reported. The risk factors associated with MSIs included body mass index (BMI) above 35 kg/m^2^ and prolonged operative time. After the implementation of the institutional prevention system in January 2017, there was a gradual decrease in the occurrence of severe events despite an increase in the number of MSIs.

**Conclusion:**

Despite the increase in the rate of MSIs, an implemented transparency and reporting system helped reduce the cases with serious consequences. To further prevent the occurrence of losing surgical elements in a surgery, we recommend educating OR staff members about responsibility and obligation to report all incidents that are caused during an operation, to develop an event reporting system as well as "rituals" within the OR setting to increase the team's awareness to MSIs.

*Trial registration* Clinicaltrials.gov (NCT04293536). Date of registration: 08.01.2021. https://clinicaltrials.gov/ct2/show/NCT04293536.

## Background

An item that is inadvertently left behind in a patient’s body during surgery or any unretrieved device needed for the surgery at each stage of the process until hospital discharge is considered a missing surgical item (MSI).

Despite the implementation of professional guidelines MSIs continue to occur. In a report from 66 countries of the 194 members of the World Health Organization, it was estimated that 312.9 million operations were carried out in 2012, with an increase of 33.6% in 8 years [1]. Some studies have reported MSI rate of 0.356/1000 patients, whereas others have reported a rate of 1/5500, with an associated mortality ranging from 11 to 35% [2, 3]. Therefore, when calculating the number of surgeries performed annually and the reported incidence of MSIs, approximately 75,665 annual cases occur, which is unacceptable [4].

One type of MSI is a retained surgical foreign body (RSFB), which applies to cases where surgical elements are forgotten in the operative field or are intentionally abandoned by the surgeon. RSFBs usually require at least a second surgery for retrieval of the lost object and carry a risk for major complications, including morbidity and death [5]. RSFBs are often underreported in order to minimize exposure to possible litigation [6]. RFSBs occur mostly in the abdominal cavity, representing more than 50% of all cases [7], and this incidence may be explained by the complexity of the abdominal cavity. RSFBs, specifically textile materials, may remain undetected many years after surgery and can lead to severe complications [8].

The Association of Perioperative Registered Nurses recommends that counts be performed before each procedure, before any cavity closure, before wound closure begins, at the end of the procedure, and at the time of any permanent changes in operating room (OR) personnel [8]. However, performing an adequate surgical count does not eliminate the risk for MSIs, as approximately 88% of cases occur when the surgical count is thought to be correct [9]. Any case of count error is preventable, which is why Medicare announced in 2007 that the program would not pay for hospital expenses in cases where counting errors occurred in operations [10].

An MSI poses tremendous mental agony and humiliation to the surgeon, in addition to possible charges of negligence; therefore, there is a need for improved systems and methods for identifying and tracking surgical items during a surgical procedure, other types of interventions are necessary, such as enhancing teamwork and communication improvement are essential for patient safety [11].

Assuta Medical Center is a private hospital that performs elective surgeries in all specialties except obstetrics and emergency surgery. In 2017 a system aimed at preventing the occurrence of MSIs was implemented at the hospital. The system included the following: (1) education of all OR workers; (2) direct responsibility of the surgeon for the surgical material; (3) responsibility of the team for maintaining a correct count of all surgical items; (4) count verification by two team members and, in the case of discrepancy, a by third responsible member who must corroborate the count and ensure its correctness; (5) in cases of material loss, the obligation to carry out a diagnosis by imaging; and (6) to report such cases in detail to the Risk Management Department, including potential cases of MSIs. All OR personnel received guidance regarding these changes in behavior and counting standards. Guidance was provided by the risk Management department to small groups of participants and in staff meetings.

The objective of this study was to examine the changes in MSI events following the implementation of the MSI prevention program.

## Materials and methods

All surgical cases registered in our medical center from January 2014 to December 2019 were retrospectively included in this study. The study was approved by the institutional ethics committee and was registered at Clinicaltrials.gov (NCT04293536). The need for informed consent was waived for this study.

An MSI was defined as any element on the surgical instrument table that was used, introduced or implanted during a surgery carried out at the institution and was unaccounted for from the moment the patient was admitted to the surgical reception room until his/her discharge from the hospital, whether or not the item was forgotten or left in the patient’s body voluntarily or was extracted before hospital discharge. RSFB was defined as cases where surgical elements were forgotten in the operative field or intentionally abandoned by the surgeon.

The outcomes of MSI were classified as unpreventable adverse events (UAEs) and preventable adverse events (PAEs). UAEs were defined as avoidable adverse medical errors that occur during the span of an operation with damage caused by medical management rather than by the underlying condition of the patient. Preventable adverse events (PAEs) were considered any event that had adverse consequences but could have been prevented without leaving any consequences.

According to the National Quality Forum of the United States, “never event” is a medical error, adverse, and serious events that are largely preventable, MSIs include “almost never events” and “never events” [12].

The conclusions of the investigation of each case enabled to identify the main member of the team who was responsible for the MSI and the person who recognized it and helped prevent a "never event".

The MSIs included in the study were classified into three groups according to severity and clinical implications. Group-1 included PAEs in which the patient’s case progressed without any type of alteration. The missing item was found without the need for any additional procedure and without consequences. Group-2 included minor consequences for the patient (reopening the operative field, prolongation of the operation or intentionally leaving the item in place), and the missing item was recognized, and the case was resolved during the operation. Group-3, defined as severe, included UAEs (RSFBs, retained broken instruments, elements left in place intentionally) with short- and long-term consequences for the patients.

### Statistical analysis

All relevant reports of MSIs used during surgery were analyzed. The characteristics of the study population at baseline were analyzed using descriptive statistics, expressed as the mean, standard deviation (SD), number and percent.

Each report was analyzed to provide information on severity, missed items, documentation of the surgical count, staff members, risk factors, and patient consequences following the event. Categorical variables were compared by chi-squared test and continuous variables were compared using *t*-test. Multivariate logistic regression analysis was performed to determine risk factors for MSI occurrence. The level of statistical significance was set at *p* < 0.05, and all *p*-values reported were 2-tailed.

## Results

### Patient demographic characteristics

During the 6-year study period, 559,910 surgeries were carried out, and 145 cases concerning MSIs were reported. The mean age of the patients was 48.67 years (SD ± 20.88), 82 (56.6%) were female (Table 1).

**Table 1:** Demographic characteristics of the patients at baseline

Characteristic	
Patients (N)	145
Age (years)	
Mean/SD	48.67 ± 20.88
Range	3–82
Gender N (%)	
Female	82 (56.55%)
Male	63 (43.45%)
Body Mass Index (kg/H^2^)	
Mean/SD	26.81 ± 6.48
Range	10.59–46.12
Operative time (min)	
Mean/SD	114.58 ± 89.24
Range	10–607
Hospital stay (days)	
Mean/SD	3.25 ± 10.78
Median	1
Range	0–123

*N* number, *SD* standard deviation, kg/H^2^ kilogram/height^2^

**Fig. 1:**
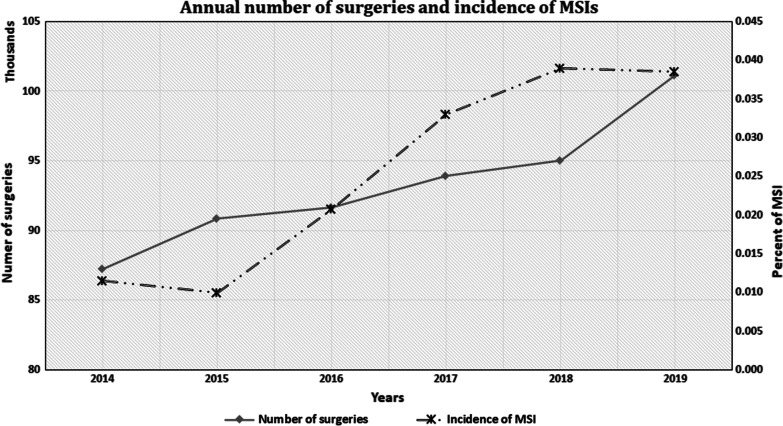
Annual numbers of surgeries and the incidence of MSI

The number of surgeries increased annually by 3.03%, which represents a 15.24% increase in the number of surgeries during the study period. The number of MSI reports increased annually by an average growth of 34.38% per year, with a total increase of 171.92% during the study period (Fig. 1). The rate of MSI in this series was 0.259/1000 operations, with a probability of 1/3888.

**Fig. 2:**
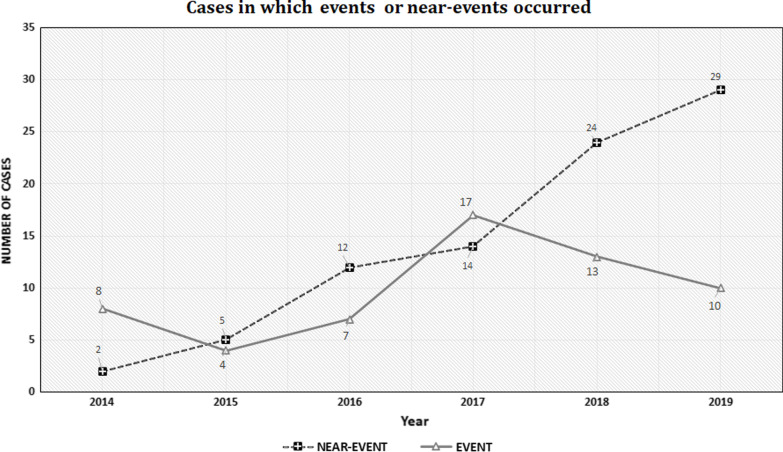
Cases in which events and near-events occurred

After the implementation of the institutional prevention system in January 2017, there was a gradual decrease in the occurrence of severe events despite an increase in the number of MSIs. PAEs increased by 73.74% annually and by 221.23% during the study period, while annual average of severe events decreased by 15.13%. In the last two years of the study, severe events decreased by 62.95% (Fig. 2).

### MSI severity

Seventy-seven cases (53.10%) were included in group-1, 47 cases (32.41%) were included in group-2, and 21 cases (14.48%) were included in group-3.

The cases in group-3, were difined as UAE, which included RFSBs, represent a prevalence of 0.037/1000 operations, and a probability of 1/26667. A total of 13 patients (8.96%) required revision due to RSFBs in the operative field, and 15 (10.34%) needed rehospitalization. Three patients developed wound infections that could have been attributable to exploration of the wound to find the lost object, and 1 patient developed severe sepsis not associated with a foreign body. There were no deaths.

### Analysis of MSIs by surgical specialty

MSIs occurred in 13 surgical specialties. Among them, 50 cases occurred in general surgery, from a total of 119,954 operations performed, representing a rate of 0.42/1000 operations. Ten of these 50 cases (20%) occurred during bariatric surgery, which was the subspecialty with the most MSI cases. Specifically, 14,767 bariatric surgeries were carried out, and cases with RSFBs represented 0.6/1000 of all bariatric surgeries. Three cases occurred during postoperative ventral hernia repair, and 1 case occurred during umbilical hernia repair. Of these cases, 11 operations were performed laparoscopically, and one was performed as open surgery.

Thirty MSIs occurred during orthopedic surgery (rate of 0.44/1000 operations), 21 during gynecologic surgery (0.34/1000 operations), 19 during otorhinolaryngology (0.27/1000 operations), and 7 during cardiac surgery (1.82/1000 operations), which was the subspecialty with the highest rate of MSIs. The rate of MSIs among the different subspecialties was statistically significantly different (*p* < 0.001).

Lower rates of MSI were observed in ophthalmology, with one MSI case in 42,881 surgeries (0.02%), otorhinolaryngology, with 19 MSI cases out of 69,921 surgeries (0.027%), and urology, with 5 cases out of 43,380 surgeries (0.12%) (Table 2).

**Table 2:** Occurrence and rate of MSI per surgical specialty

Surgical specialty	Surgeries	MSIs	Rate	*p* value
Open cardiac-surgery	3855	7	1.82‰	< 0.001*
General surgery	119,954	50	0.42‰	
Gynecology	61,734	21	0.34‰	
Ophthalmology	42,881	1	0.02‰	
Orthopedic surgery	69,827	31	0.44‰	
Otorhinolaryngology	69,621	19	0.27‰	
Urology	43,390	5	0.12‰	

Cardiac surgery is the specialty with the higher rate of MSIs, and Ophthalmology is the specialty with the lower rate of MSIs. The rate of occurrence is expressed per thousand of surgeries

*MSIs* Missed surgical items

^*^Chi-square test, significant for differences in MSI occurrences between surgical specialties, *p* ≤ 0.05

### Retained MSIs

The most common RSFBs were textile items such as gauze sponges, gauze and round stick sponges, which occurred in 42 cases (28.97%). Suture needles were the cause of 41 cases (28.28%), of which 22 (53.65%) occurred during laparoscopic surgery or arthroscopy. Seven patients (4.83%) were discharged from the hospital with a vein-cannula left in place. There were five cases (3.45%) of drill tip breaks during surgery, and in two cases, the broken tips were left in the bone. Ten more cases were caused by loss of a wire, a scalpel, a clip marker, drains and gloves (Table 3).

**Table 3:** Type of devices causing the incident and its severity

Device	Consequence						Total	
	Severe		Mild		No			
	N	%	N	%	N	%	N	%
Textile	9	42.86	13	27.66	20	25.97	42	28.97
Needle	2	9.52	13	27.66	26	33.77	41	28.28
Instruments	4	19.05	8	17.02	18	23.38	30	20.69
Clip marker	1	4.76	2	4.26	1	1.30	4	2.76
Drain	2	9.52					2	1.38
Dental prosthesis	1	4.76					1	0.69
Vein-cannula	1	4.76	2	4.26	4	5.19	7	4.83
Bougie	1	4.76					1	0.69
Wire			2	4.26	3	3.90	5	3.45
Tourniquet			1	2.13			1	0.69
Scalpel			1	2.13	2	2.6	3	2.07
Syringe			1	2.13			1	0.69
Globe			1	2.13			1	0.69
Drill			3	6.38	2	2.60	5	3.45
Trocar valve					1	1.30	1	0.69
Total	21	14.48	47	32.41	77	53.10	145	

*N* number, *%* percent

### The clinical setup where the MSIs occurred

Among the 145 cases of MSIs analyzed, in 120 cases the missing item was suspected while the patient was still in the OR, in 94 cases (78.33%) the surgical count was abnormal, which led to the appropriate actions to find the missing item, in 22 cases (18.33%) the count was wrongly considered normal, in 2 cases the count report was lost, and in 2 cases a count was not carried out.

In 10 cases patients were sent home without removing a venous access or removing hemostatic tampons from the nose or vagina. One patient after cardiac surgery was sent home with pacemaker electrodes retained after cardiac surgery. Seven cases occurred during hospitalization: in two cases a Jackson-Pratt drain was lost in the abdominal cavity while being removed, requiring reintervention, and one case a bougie used for calibration was forgotten in the esophagus after bariatric surgery and was not included in the inventory. Two MSI cases, which occurred in the in vitro fertilization department, textile material was forgotten in the vagina. In two additional cases, which occurred in the recovery room, one nasal tampon and one dental prothesis were missed.

Twenty-three cases (15.86%) were not reported by any team member to the hospital’s Risk Management Department.

### Risk factors for MSIs

Multivariate logistic regression showed that body mass index (BMI) above ≥ 35 kg/m^2^ was associated with an increased risk of MSI (*p* = 0.005) (Table 4). Fourteen patients had a BMI ≥ 35 kg/m^2^. Among these, 10 cases occurred during bariatric surgery, 3 cases occurred during postoperative ventral hernia repair and one case—during umbilical hernia repair. Eleven operations were performed laparoscopically, and one was an open surgery.

**Table 4:** Risk factors for MSI

BMI	N	Mean BMI	SD	*p* value
Under 20 kg/m^2^	18	16.23	± 2.579	0.228
20.1–27 kg/m^2^	58	23.82	± 1.577	0.128
27.1–35 kg/m^2^	57	27.09	± 2.253	0.288
Above 35 kg/m^2^	12	40.09	± 3.600	0.005

Operative time	N	Mean op.time	SD	*p* value
60 min. or less	41	34.83	± 15.26	< 0.001
61 to 120 min	52	90.12	± 18.26	< 0.001
121–180 min	25	143.97	± 16.72	0.012
181 min. or more	27	254.67	± 94.29	< 0.001

The variables that increase de risk of MSIs are presented in the table, others variables as team changes, blood loss, sudden complications, were analyzed and did not show differences in the results

*N* Number of cases, *BMI* Body mass index, *SD* standard deviation, *Op.* operative

Analysis of MSI risk by operative time (less than 60 min, 61 to 120 min, 121 to 180 min and more than 181 min), showed significant differences among operative time groups (*p* < 0.001), with higher risk of MSI in longer operations.

Although there were 17 cases (14.17%) of personnel change during surgery, the involvement of more than one surgical team did not increase in the risk for MSIs. In addition, severe intraoperative complications occurred in 13 cases (10.83%;10 cases of massive bleeding and three cases of diffuse peritonitis with extensive peritoneal lavage) without an increased risk for MSIs.

### The team involved in the operation

A total of 123 surgeons, all of them males, were involved in the 145 MSI cases (a rate of 1.18 cases per surgeon). Eleven surgeons were involved in 2 MSI events, four—in 3 MSI events, and one surgeon was involved in four MSI events. The analysis showed that a team member may have primarily prevented the occurrence of MSIs. Surgeons could have prevented 102 MSIs (70.30%), and nurses could have prevented 24 MSIs (16.55%). In six cases (4.14%), which included instruments or textile packages with incorrect content, the responsibility for the MSI was attributed to the manufacturer, while in seven cases (4.83%), which included instruments in poor condition, the responsibility was attributed to hospital administration. In other cases, the responsibility was attributed to technicians, patients, and to an indeterminate source (Table 5).

**Table 5:** Member of the team or entity involved in MSI

Factor	Attributable			Prevent		
	N	Percent	*p* value	N	Percent	*p* value
Surgeon	102	70.34%	< 0.001*	16	11.03%	< 0.001*
Nurse	24	16.55%	0.469	96	66.21%	< 0.001*
Device company	7	4.83%	0.002*	0	0%	–
Hosp. Adm	7	4.83%	0.002*	0	0%	–
Technician	1	0.69%	< 0.001*	0	0%	–
Patient/family	2	1.38%	< 0.001*	3	2.07%	< 0.001*
Undetermined	2	1.38%	< 0.001*	30	20.69%	0.278

In 70.34% of MSIs cases, the responsibility falls on the attending surgeon, such as breaking a suture needle and leaving it abandoned in the tissues or breaking a drill in the bone. In 24% of MSIs cases, the responsibility is attributable to the scrub nurse, such as the erroneous count of elements. An instrument that does not work properly can be attributed to the company that produces it or to the hospital administration in case of poor maintenance. Likewise, according to each case, it is possible to verify the member of the involved team that prevented a case of MSI from occurring, in these cases 66.21% is attributable to the scrub nurse or circulating nurse

*N* Number, *Hosp. Admin.* hospital administration, *MSI* missed surgical items

*t test significant for differences between MSI-preventing and attributable team members *p* ≤ 0.05

## Discussion

Performing surgery involves the use of numerous objects arranged on the instrument table. These elements can accidentally be lost in the operative field or around the operating table. An estimated 1,500 operations result in MSIs each year in the United States, resulting in substantial morbidity [13].

The number of operations increases every year. Weiser predicted a 33% increase in surgeries in 10 years [1]. Analysis of the surgeries performed at our hospital showed a registered increase of approximately 15% in 6 years, with an increase in more complex surgeries in each specialty and considering the fact that only elective surgeries are performed. Previous studies have recognized that emergency surgeries and surgery with sudden changes are at higher risk for MSIs [6, 14]. With the increase in the number of surgeries performed, the number of MSIs has also increased, coinciding with Mehtsun et al. [14], who stated that MSIs are still common despite new surgical techniques and equipment. Furthermore, the number of MSIs reported has increased despite the application of a checklist for patient safety, as recommended by several authors [15–18].

Elsharydah et al. have reported a MSI rate of 13/100000 cases [19], while our results showed a prevalence of 25.9/100,000, which is almost double the reported value. Our probability result of 1/3888 cases is much higher than that reported by Stawicki, namely, 1/6975 cases [20]. Nevertheless, the prevalence of severe consequences for RSFBs is similar to that reported by Gunnar et al. of 1/23908 cases [21]. As we have shown, serious events decreased in the last 2 years of the study by 62% following the improvement in “never-events” prevention according to Fencl's guidelines [22].

Fencle’s guidelines include team responsibility, reducing the effect of distractions, noise, and interruptions, employing consistent counting methods, standardized processes for reconciling discrepancies, and continuous quality improvement [22]. According to our analysis, “never events”, such as the involuntary retention of a foreign object in an operative field after surgery, represented 17.21% of the events that occurred in the OR, similar to the 14.40% reported in The United States in 2018 [23].

Israel's Ministry of Health has issued a circular that defines how to carry out the count of items in surgery and assigned the responsibility of the count to the attending surgeon [24]. Even so, there were elements that were not included in the count, such as bougies used by anesthesiologists. Following a case of a forgotten bougie in the esophagus, the hospital added elements used by anesthesiologists to the count. Considering that most medical errors are a result of fallible humans working in chaotic, unpredictable, and complex clinical environments, we agree with Agrawal et al. that individual accountability must be balanced with system improvement [25]. It is understandable that human errors do occur; however, they must be deemed unacceptable, and every effort must be made to prevent them.

Similar to our findings, it is well recognized in the literature that most RSFBs occur in abdominal surgery [26–28]. RSFBs in the abdomen and pelvis were reported in more than 50% of cases [29]. Our results are in discordance with Steelman et al., who reported that 25.9% of MSI cases occurred in obstetrics and gynecology [29], but this difference may be attributed to the fact that our medical center does not provide obstetric services or performs emergency surgery. Furthermore, in our study, we found that 19cases out of 69,921 surgeries (0.027%) of MSIs occurred in otorhinolaryngology, differing from Steelman, who reported an incidence of 0.4%; in the rest of the specialties, our results were similar to those reported by that author [29]. The specialty with the highest incidence of MSIs was thoracic surgery, coinciding with previous reports [30, 31]. Abe et al. described 68 MSI cases in thoracic surgery, which represents 9.1% of adverse events [32]. The high incidence of MSIs in thoracic surgery can be attributed to reasons such as surgical complexity, team stress and operative time.

Textile elements were the most frequent cause of MSIs, which was in accordance with the literature. In our study, 28.97% of MSIs were textile elements, although this proportion was lower than the rate of 52% and 43%, previously reported by Lincourt [33] and Greenberg [34], respectively. New technologies using radiofrequency tracking devices that were developed for counting and finding textiles [35], reduced the number of MSI cases by 93% compared with 77% reduction by traditional counts [36]. Despite the availability of many such devices, according to Coustasse, their significant total expenses and unclear return on investment explain their lack of acceptance [37].

MSIs involving needles occurred in 28.28% of cases. Among these cases, more than 50% occurred during minimally invasive surgery, especially when the needles were passed through the trocar. In a study that included 305 minimally invasive surgeons from 11 specialties, 63% reported experiencing problems with lost needles [38]. The problem of lost needles in the operative field increases with smaller size. A 17-mm needle can be seen on X-ray by 84% of surgeons, while only 13% of them would be able to identify a 13-mm needle on an X-ray projection [39].

A fifth of the MSI cases in the present study (20.69%) was due to forgotten instruments, which may be easily prevented by simple counting. Instrument count prevented oversight in 66% of cases; an incorrect normal count leads in many cases to consequences that can be serious. Egorova et al. was the first to quantify the diagnostic accuracy of counting, and defined a counting sensitivity specificity of 77.2% and 99.2%, respectively. However, the positive predictive value was only 1.6% [40]. To date, no system has been developed that excludes counting and the human factor from the instrument inventory. Radiofrequency identification systems designed to count and find textiles are not applicable to instruments and needles.

The surgical count process stands out among the practices advocated by the World Health Organization to ensure surgical safety [41]. During surgery, the surgeon should not unquestioningly accept correct count reports but should develop the habit of performing a brief but thorough routine post-procedure wound/body cavity exploration before wound closure [42].

More than 76% of forgotten items during surgery occurred due to nurses. Miscounts trigger use of the Incorrect Count Safety Checklist, which can be used to determine whether a count completed at the procedure’s conclusion is consistent across disciplines (circulating nurses, scrub nurse, surgeons) [13]. Individual accountability and effective teamwork can help ensure patient safety [43]. While inappropriate staff behavior during surgical procedures can disrupt surgical performance and compromise patient safety [44], which can be confirmed in our study in cases where the count was not carried out or the report was lost. Communication failure in the OR was reported to occur in approximately 30% of team exchanges, with a third resulting in effects that compromised safety [45]. Other factors that could affect teamwork are fatigue, not having worked together with team members, unfriendly relationships between members, and unfamiliar work environments.

Interestingly, the surgeons involved in MSIs were all male, suggesting that the risk for MSI may be related to gender. Differences between male and female surgeons have previously been described. Wallis has found that 30-day mortality was significantly lower in patients treated by 774 female surgeons compared to those treated by 2540 male surgeons, without significant differences in the characteristics of both groups of patients [45]. The surgeon's ego causes more disruptive attitudes in men than in women during procedures in the surgical theater [46]. Female surgeons were described as having significantly different personality profiles than male surgeons [47], and being significantly more extroverted and agreeable than male surgeons relative to the nonsurgical population means [48].

Similar to previous reports [49], we have found that obesity, specifically, BMI above 35 kg/m^2^, was a risk-determining factor for retaining surgical articles. In contrast, Moffatt-Bruce et al., did not find relationship between BMI and the risk for retention of surgical items [50]. In this study, the incidence of RSFBs during bariatric surgery was 24% greater than that during general surgery. We did not find any literature on RSFBs in bariatric surgery, only cases of incomplete gastric band extraction as reported by Cattanach et al. [51]. Bariatric surgery involves the use of multiple trocars, a high number of instruments and sutures, to which must be added the challenges of obesity.

Longer operations were associated with greater risk for MSI. Judson et al. affirmed that increased case duration was strongly associated with an increased risk of a miscount [13], which may explain the risk of MSI occurrence due to fatigue and the increased number of sponges and instruments used. In a study performed by Barger, it was concluded that extended-duration work shifts were associated with an increased risk of significant medical errors, adverse events and attentional failures in interns across the United States [52].

Personnel changes and operative complications did not increase the risk of MSIs, as has been stated by other authors [49, 51]. In contrast, Lincourt et al. found significant effects of personnel changes during the operation [33].

Currently several steps are being used to reduce the occurrence of lost or retained surgical items. These include continuous instruction and training about critical procedures to ensure patient safety such as "Time out" procedure and OR counting procedure, performing independent timely observations on the knowledge and compliance of the relevant procedures in the OR by the its staff. These observations provide opportunities to instruct the staff on possible "clashes" in the operation room (e.g., between the physician and the OR nurse), and to reinforce the importance of communication among the OR team during work. Cases and examples are discussed with the OR staff in order to implement a culture of learning from errors, and to obtain broad insight on working procedures. If relevant, a root cause analysis is performed.

### Implications for practice

To further prevent the occurrence of losing surgical elements in a surgery, we recommend educating OR staff members about responsibility and obligation to report all incidents that are caused during an operation. In addition, an event reporting system should be developed, which would make use of clear forms that are easy to complete and take a short time to do so. A mandatory x-ray method to identify missing elements should be developed, and a methodical inventory count of the elements used in the surgery should be performed by personnel involved in the operation as well as by uninvolved personnel. We also believe that there is a crucial importance for "rituals" within the OR setting. These include "speak up" and "listen up" techniques as a tool for increasing the team's awareness and highlighting the importance of the topic. Finally, we believe that every health organization should promote the crucial role of its medical teams in maintaining the safety and quality of care—not only for the sake of the patients but also to improve teams’ engagement and to prevent the second victim phenomenon.

The results of this study objectively present the MSI cases that occurred in the institution. The study findings are limited by the low percentage of MSI cases, and prevent drawing definitive conclusions. Long-term follow-up of the patients in whom items were left in the operative field at the hospital is warranted.

## Conclusion

This study demonstrated the incidence of events in which surgical elements were missing or forgotten during an operation, as well as the prevalence of cases with consequences for the patient ("never events"). Despite the increase in the rate of MSIs, an implemented transparency and reporting system helped reduce the cases with serious consequences. Cardiac surgery was the specialty with the highest incidence of events. Textile elements were the most frequently retained items. Patient BMI ≥ 35 kg/m^2^ and prolonged operations were risk factors for RSFBs. Coordinated teamwork, education and reports of events help to avoid MSI occurrence.

## Data Availability

The datasets used and/or analyzed during the current study are available from the corresponding author on reasonable request.

## Abbreviations

MSI: Missing surgical item
RFSB: Retained surgical foreign body
UAE: Unpreventable adverse events
PAE: Preventable adverse events
